# Acute-on-Chronic Valproic Acid Overdose: A Toxicology Simulation Case for Medical Students

**DOI:** 10.15766/mep_2374-8265.11649

**Published:** 2026-11-05

**Authors:** Grant Thesing, Bradley Sheffield, S. Jennifer Wang, Deron Tyler, Michelle Troendle

**Affiliations:** 1 Medical Student, Virginia Commonwealth University School of Medicine; 2 Medical Student, Virginia Commonwealth University School of Medicine; 3 Medical Student, Virginia Commonwealth University School of Medicine; 4 Medical Student, Virginia Commonwealth University School of Medicine; 5 Associate Professor, Department of Emergency Medicine, Virginia Commonwealth University Health (VCU Health); Attending Physician, Division of Clinical Toxicology, VCU Health

**Keywords:** Valproic Acid, Overdose, Simulation, L-Carnitine, Emergency Medicine, Internal Medicine, Toxicology

## Abstract

**Introduction:**

Overdose of valproic acid, an antiepileptic medication prescribed for seizure disorders, bipolar disorder, and migraine prophylaxis, is common but underrepresented in medical school curricula. Because valproic acid is widely prescribed and toxicity can occur at both therapeutic and supratherapeutic doses, medical students need a foundation in recognizing and managing it. To close this gap, we built a multimodal educational intervention combining didactic instruction with an experiential simulation.

**Methods:**

Fourth-year toxicology elective students completed a knowledge pretest and a confidence-based Likert questionnaire before attending a lecture, then worked in small groups through a 15-minute simulated encounter with a patient with acute-on-chronic valproic acid overdose, followed by a 15-minute debrief. The scenario's critical actions comprised a focused history, neurologic exam, valproic acid and ammonia levels, L-carnitine administration, and toxicology/psychiatry consultation; required materials were a manikin, intravenous supplies, and pharmaceuticals. A posttest and follow-up Likert questionnaires captured knowledge change, reactions, and intended practice change.

**Results:**

Across 10 sessions, 48 students completed both tests; scores rose from a mean of 58% (*SD* = 21%) to 97% (*SD* = 6%) (Wilcoxon *W* = 0.0; *P* < .001), and every group completed the critical actions checklist. Confidence in all four domains (identifying manifestations, obtaining diagnostic studies, interpreting diagnostic studies, and initiating treatment) rose from a median of 3-4 to 5 (Mann-Whitney *P* < .001 for all). Reactions to the workshop were favorable, and most learners reported intent to change practice.

**Discussion:**

This multimodal simulation was an effective way to teach valproic acid overdose management.

## Educational Objectives

By the end of this activity, learners will be able to:

1. Describe the pharmacokinetics and toxicokinetics of valproic acid.
2. Obtain a focused toxicologic history, and perform a targeted physical and neurologic examination for a simulated patient with valproic acid overdose.
3. Interpret clinical findings and laboratory results associated with valproic acid toxicity.
4. Implement evidence-based management steps for valproic acid overdose using a critical actions checklist, including administration of L-carnitine and consultation with appropriate services.

## Introduction

Valproic acid is an antiepileptic medication used as both monotherapy and adjunctive therapy to treat simple, complex-partial, and complex-absence seizures.^1^ Additionally, it is prescribed off-label for managing bipolar disorder, migraines, diabetic peripheral neuropathy, postherpetic neuralgia, and behavioral disturbances, including impulsivity, agitation, and aggression.^1^

Valproic acid overdose is a significant concern in the United States, with thousands of cases reported annually.^2^ Symptoms of overdose include hyperammonemia, hepatotoxicity, bone marrow suppression, tremors, seizures, cerebral edema, and death.^3^ Complications can also arise at therapeutic doses because metabolites may suppress the urea cycle, leading to elevated ammonia levels and encephalopathy.^1^ Given valproic acid's widespread use, potential toxicity, and high overdose rates, it is essential for medical students to develop a strong foundation in managing both patients who may have adverse clinical effects at therapeutic doses and patients who have overdosed.

We designed a multimodal educational intervention—didactic instruction paired with experiential simulation^4^—grounded in Kolb's experiential learning theory,^5^ which holds that knowledge is best constructed through concrete experience followed by reflective observation. A preceding lecture ensures baseline pharmacologic knowledge, while the simulation and debrief let learners apply and reflect on that knowledge in a psychologically safe, structured environment—addressing skills (history taking, physical exam, procedural skills, clinical reasoning, and escalation of care) that didactic teaching alone cannot build. This case maps to four Association of American Medical Colleges (AAMC) Core Entrustable Professional Activities (EPAs) for Entering Residency:^6^ EPA 1 (gather a history and perform a physical examination), EPA 3 (recommend and interpret diagnostic tests), EPA 10 (recognize and initiate management for a patient requiring urgent care), and EPA 12 (perform general procedures, including intravenous [IV] placement). Toxicologic emergencies are well suited to this approach because safe, hands-on practice with an acutely poisoned patient is rarely available in clinical rotations. We previously piloted this lecture-simulation-debrief structure for another toxicologic emergency,^7^ and adapted it here to address the specific curricular gap in valproic acid overdose management. To our knowledge, there are no other simulated cases of valproic acid overdose within *MedEdPORTAL*. We acknowledge a previously published pediatric valproic acid toxicity simulation in the Toxicology section of the American College of Emergency Physicians website by Gordon et al.^8^ Our simulation differs in several key ways: it features an adult rather than a pediatric patient, represents a less critically ill presentation that is more appropriate for medical student learners, involves an acute-on-chronic overdose rather than a purely chronic exposure, and includes a structured review of how to obtain a comprehensive toxicologic history.

## Methods

### Activity Design and Development

We designed this activity as a lecture-simulation-debrief sequence for fourth-year medical students rotating through the clinical toxicology elective at Virginia Commonwealth University School of Medicine (VCUSOM). The senior author, a board-certified clinical toxicologist and the elective director, developed the case, drawing on prior experience creating a tetanus simulation with the same structure.^7^ We used the Understanding by Design framework to align the lecture, simulation, debrief, and assessments with the learning objectives.

### Curricular Context and Learner Preparation

The activity took place during the third week of a 4-week clinical toxicology elective; the broader intended audience is medical students during their clinical years of training. A faculty facilitator is required, ideally trained in emergency medicine, internal medicine, or neurology given alignment with the case's clinical content, and trained on the simulation manikin and software to accurately reflect changes in the patient's condition. Required student preparation includes basic airway management and peripheral IV placement skills, familiarity with overdose-focused history and physical exam elements, and prior completion of the valproic acid lecture.

### Equipment and Environment

We delivered the lecture in a standard classroom at VCUSOM, with students facing a projection screen and the facilitator facing the students.

We conducted the experiential learning component in a simulation lab designed to replicate an emergency department, organized into stations (eg, pharmaceutical, IV access, and control panel); most equipment, though not strictly required for the case, was included to enhance realism (Appendix A).

We programmed the manikin to exhibit tachycardia with occasional sobbing; the facilitator connected voice controls to portray a confused patient and the patient's significant other. Participants viewed a cardiac monitor throughout, with both cardiac monitor and manikin responses adjusted in real time based on students’ interventions.

### Personnel

The simulation required a facilitator, who portrayed multiple roles—the patient's voice, the patient's significant other, a nurse, and a consultant—and adjusted the manikin's settings in response to student actions. We did not use actors or standardized patients. This single-facilitator design reflects our institution's staffing model. Programs with additional staff could instead assign a dedicated simulation technician to operate the manikin, separate from a confederate portraying the patient and consultants, reducing facilitator task switching.

### Implementation

Two weeks before the lecture and simulation, students completed a pretest (Appendix B) and a Likert scale questionnaire (Appendix C) to establish baseline knowledge and confidence; we did not provide pretest answers.

The day before the simulation, 66 students attended a one-hour lecture on valproic acid (Appendix D). The following day, students who volunteered to participate actively worked in groups of 4-6 through a single simulated case involving a 26-year-old patient with acute-on-chronic valproic acid overdose; the remaining students observed the simulation and attended the debrief. Within each group, students self-organized into five resuscitation-team roles—team leader, airway, IV access, pharmacy, and scribe, with students in smaller groups covering more than 1 role—and interviewed the patient and significant other, performed a physical exam, initiated management, requested labs, provided treatments, and determined disposition; we did not require admission orders or an encounter note.

We prepared the room per Appendix A and configured the software for the simulated case (Appendix E), displaying requested labs and imaging on a television screen (Appendix F). We used a critical actions checklist (Appendix G) and allowed 15 minutes per case.

Immediately after, we led a 15-minute structured debrief (Appendix H). Students then completed an identical posttest (Appendix I) and Likert questionnaire (Appendix J), and we reviewed posttest answers with explanations afterward.

### Debriefing

We divided the 15-minute debrief into two phases aligned with the Plus/Delta framework. In the reflective phase (Section I), learners sat in a semicircle facing the facilitator, who posed open-ended questions inviting the team to identify what went well (“Plus”) and what could be improved (“Delta”), letting students lead discussion of their own clinical reasoning and teamwork before facilitator input. The facilitator then guided a content-focused review across six sections aligned with the case objectives: general toxicology principles, including history taking and methods to decrease absorption or enhance elimination (Section II); valproic acid pharmacokinetics and pathophysiology (Section III); pertinent history, exam, and laboratory findings and their relationship to the drug's mechanism of action (Section IV); treatment, consultation, and disposition (Section V); alternative clinical presentations (Section VI); and take-home points, including pregnancy category and other laboratory aberrancies (Section VII). Throughout, the facilitator asked learners to justify why they pursued or deferred specific actions, linking content review to clinical reasoning. Because valproic acid toxicity is infrequently encountered clinically, we prioritized this structured review to consolidate baseline knowledge alongside the reflective discussion (Appendix H).

### Assessment

We evaluated the activity using a single-group pretest-posttest design. Participation in the evaluation was voluntary and did not affect elective grades, and the facilitator did not evaluate or report individual performance for grading. Learners completed the pretest 2 weeks before the activity to minimize recall bias. The assessment was multifactorial and mapped to the New World Kirkpatrick Model,^9^ under which self-reported confidence and intent to change practice—not only knowledge—are classified as Level 2 (Learning) outcomes. We measured knowledge acquisition by comparing pretest and posttest scores (Appendices B and I). The seven-item multiple-choice test addressed indications, mechanism of action, and metabolism of valproic acid, as well as abnormal laboratory values and management of overdose; items were equally weighted, with scores reported as percentages. All authors contributed to item development, and an emergency medicine physician with fellowship training in medical education reviewed the questions for clarity and alignment with learning objectives. The assessment instrument was not formally validated. Participants completed the same assessment after the simulation and debrief to measure knowledge gains.

We used a critical actions checklist (Appendix G, Level 2: Learning) to evaluate performance, capturing essential clinical actions expected during initial evaluation and management: history of present illness, focused physical exam, fingerstick blood glucose, electrocardiogram (ECG), IV access (credited for requesting or attempting access regardless of technical success), valproic acid and ammonia levels, L-carnitine, specialty consultation, and hospital admission.

We evaluated participant reactions to the workshop using a Likert scale questionnaire (Appendix J, questionnaire 1), administered immediately after the simulation to assess perceived fit to training level, degree of challenge, workshop length, and value of practicing procedural skills.

A second questionnaire (Appendix C, and Appendix J, questionnaire 2), administered before and after the simulation, measured confidence in identifying clinical manifestations, obtaining and interpreting diagnostic studies, and initiating treatment for valproic acid overdose.

A third postsimulation questionnaire (Appendix J, questionnaire 3) assessed intended practice changes and anticipated transfer of skills, including history taking, physical exam, diagnostic study selection, and procedural skills, to other clinical contexts.

These domains aligned with the overall learning objectives, and we based this format on prior validation of similar self-reported instruments. Students could also submit anonymous narrative feedback.

## Results

Between June 2025 and March 2026, 66 fourth-year medical students rotated through the clinical toxicology elective. All 66 students attended the lecture; of these, 48 (73%) completed both the pretest and posttest, 43 (65%) completed the presimulation Likert scale questionnaire, and 35 (53%) completed the postsimulation Likert scale questionnaire. We conducted the simulation 10 times; 57 students actively participated in the simulation, and 9 observed it.

The mean pretest score was 58% (range = 14%-100%; *SD* = 21%), and the mean posttest score was 97% (range = 71%-100%; *SD* = 6%), as shown in Figure 1. A Wilcoxon signed-rank test demonstrated a statistically significant improvement in scores (*W* = 0.0; *Z* = −5.91; *P* < .001), with a large effect size (*r* = 0.853).

**Figure 1.**
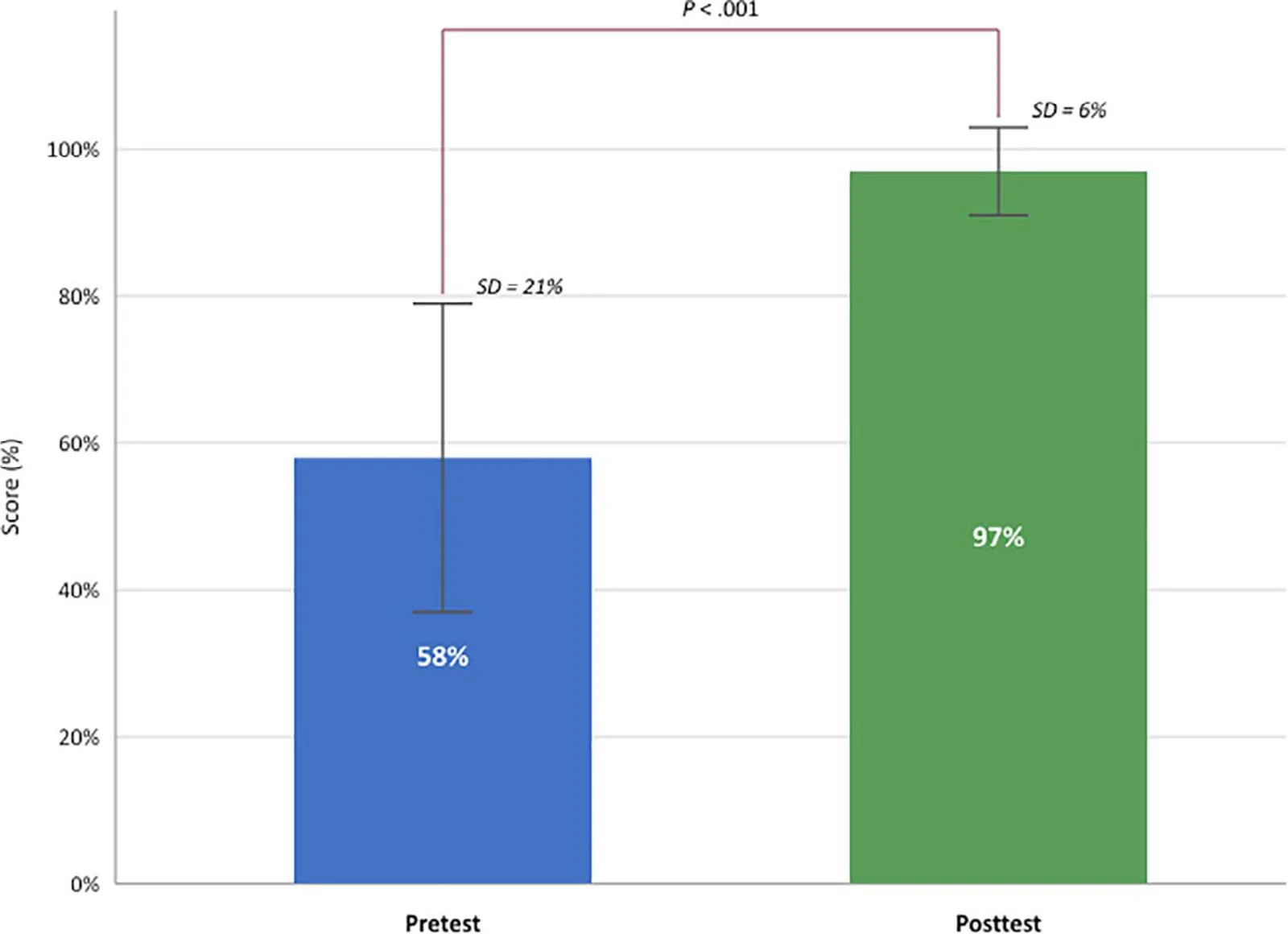
Mean pretest scores (58%; range = 14%-100%; *SD* = 21%) and posttest scores (97%; range = 71%-100%; *SD* = 6%); *N* = 48. Error bars represent ± 1 *SD*; statistically significant difference (*P* < .001) on Wilcoxon signed-rank test (*W* = 0.0; *Z* = −5.91), with large effect (*r* = 0.853).

All groups completed all critical actions within 15 minutes. Although some teams required occasional prompts or guidance, as outlined in Appendix E, all groups ultimately completed every required checklist component.

Median presimulation confidence scores on the Likert scale questionnaire for identifying clinical manifestations, obtaining and interpreting appropriate diagnostic studies, and initiating treatment were 3, 4, 4, and 3, respectively; all increased to 5 following the simulation. Because surveys were anonymous and not paired by student, we applied a Mann-Whitney U test to analyze pre- and postsimulation data. All four domains demonstrated statistically significant improvement (*P* < .001 for all). These results are summarized in Figure 2.

**Figure 2.**
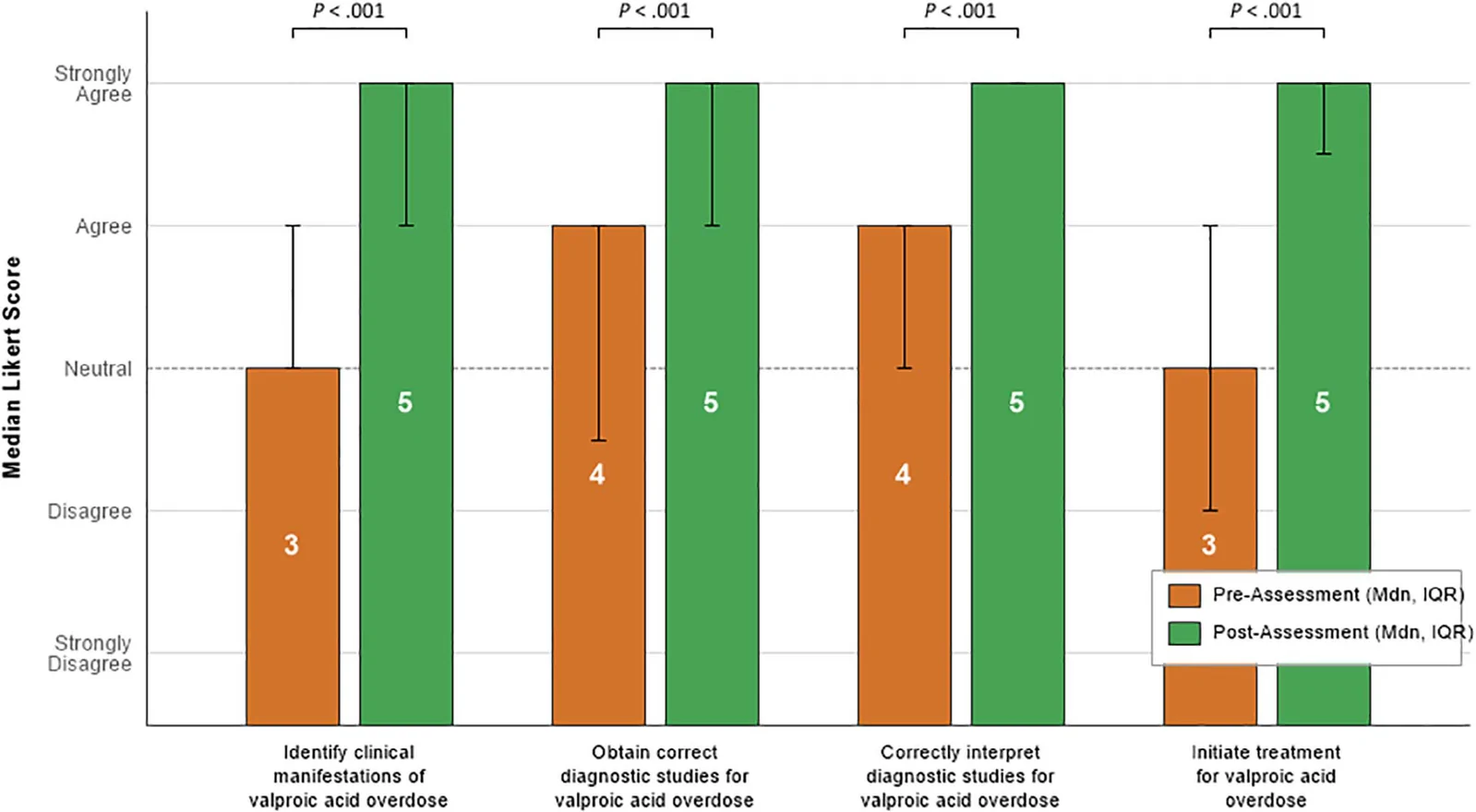
Median presimulation (*N* = 43) and postsimulation (*N* = 35) responses on perceived ability to identify the clinical manifestations, order and interpret diagnostic studies, and initiate management for a valproic acid overdose, with interquartile range (IQR; error bars represent IQR [quartile 1–quartile 3]).

On the Likert scale questionnaire assessing reactions to the workshop, students rated the simulation experience favorably. The majority of respondents agreed or strongly agreed that the simulation was appropriate for their level of training (strongly agree: 69%; agree: 29%), that they felt challenged by the case (strongly agree: 63%; agree: 34%), that the length of the workshop was appropriate (strongly agree: 74%; agree: 26%), and that they appreciated the opportunity to perform procedural skills (strongly agree: 68%; agree: 21%). These findings are summarized in Figure 3.

**Figure 3.**
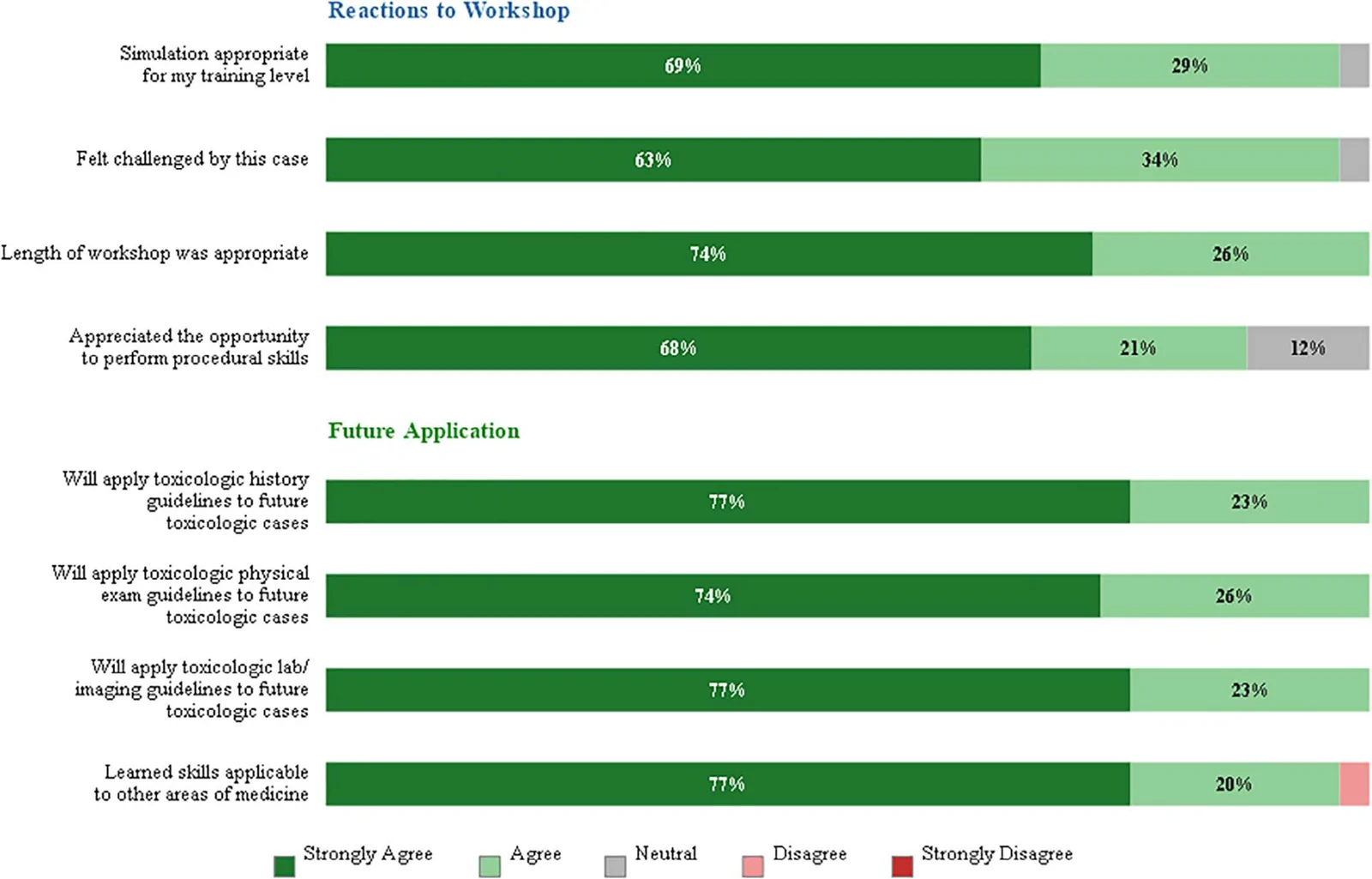
Postsimulation responses (*N* = 35) on reactions to workshop and intent to apply skills obtained to future practice. Bars show percentages of responses per category.

On the Likert scale questionnaire assessing intent to change practice, students expressed a strong intention to apply the toxicologic frameworks in future clinical settings. All learners agreed or strongly agreed that they would apply toxicologic history-taking guidelines (77% strongly agree, 23% agree), toxicologic physical examination principles (74% strongly agree, 26% agree), and laboratory and imaging guidelines (77% strongly agree, 23% agree) in future clinical settings. The majority of students reported that the skills practiced during the workshop were transferable to other areas of medicine (77% strongly agree, 20% agree). These findings are summarized in Figure 3.

In their narrative feedback, learners reported that the simulation effectively delivered the core content and improved their confidence in recognizing and managing valproic acid toxicity. Several participants specifically noted that the case reinforced their ability to identify clinical manifestations of valproic acid overdose and to initiate appropriate treatment, including the use of L-carnitine for valproic acid–induced hyperammonemia. Learners appreciated the opportunity to practice a structured toxicologic history and physical examination, noting that the simulation reinforced general toxicology concepts applicable to a broad range of overdose presentations. They also valued the chance to practice procedural skills, particularly peripheral IV access and airway assessment in the setting of a patient with altered mental status and secretions. Lastly, learners identified IV placement, airway management, ECG interpretation, and toxicologic history-taking and physical exam skills as transferable competencies they intend to apply to future patients presenting with overdose or toxicologic emergencies.

Currently, the senior author is the sole user of this simulation. Although we did not conduct formal evaluator training, evaluators should be familiar with the critical actions checklist prior to implementing the simulation. Because participation was voluntary and without impact on grades, we have not developed a formal remediation process.

## Discussion

We designed this simulation to present students with the opportunity to manage a real-life example of valproic acid overdose in a safe learning environment. We aimed to deepen their medical knowledge regarding the drug's pharmacokinetics and available treatment options, and fine-tune their approach to the poisoned patient. Statistically significant improvements in test scores and perceived ability to manage these patients, together with favorable reactions and reported intent to change practice, suggest the intervention achieved its intended objectives though our single-arm design cannot isolate the simulation's specific contribution from the broader educational intervention (see discussion of limitations further in this section).

On reflection, the simulated patient arrived 10 hours after ingestion, rendering single-dose activated charcoal ineffective; we addressed this with hypothetical debrief scenarios where it would have been appropriate. Although the patient was not critically ill, we discussed seizure risk and benzodiazepine management, and the potential for hypotension requiring vasopressor support, during debrief. Because this acute-on-chronic presentation does not represent all clinical manifestations of valproic acid toxicity, we also discussed how body burden and carnitine stores can drive variability. Despite the facilitator managing multiple roles throughout each session, students did not report or exhibit noticeable confusion about which role was being portrayed at a given moment. We also noted that participants’ focused histories were sometimes incomplete despite establishing the ingestion; a pre-brief highlighting key history questions (substance, dose, co-ingestions, timing, and intent) could strengthen this further.

We acknowledge several limitations. This single-center study had a relatively small sample size, limiting generalizability and statistical power. Implementation required substantial time, personnel, and access to a simulation center, which may limit its feasibility elsewhere, and the scenario is most relevant to learners in emergency medicine, internal medicine, toxicology, and neurology. Because participation required voluntary enrollment in a toxicology-focused elective, our sample likely reflects students already inclined toward related specialties. This single-arm design also lacks a comparison group, so gains cannot be attributed specifically to the simulation rather than to the broader educational intervention; we are addressing this in an ongoing comparison of simulation-based and lecture-only cohorts in our 2-week online elective. We did not systematically record the number or type of prompts required per group. The multiple-choice test was not formally validated. Although pretest and posttest knowledge assessments were paired, Likert scale responses were not linked at the individual level; a unique identifier would have enabled direct comparison of participants’ perceptions before and after the simulation. Survey response rates were also lower than for the knowledge assessments, likely reflecting the different administration platforms (Canvas vs. Google Forms), and the Likert scale survey measured self-reported perceptions rather than objective performance, introducing possible response bias. Because the pretest was administered 2 weeks before the lecture and simulation, some students may have independently reviewed material in the interim; and because the posttest was identical to the pretest, score gains may partly reflect a test-retest effect rather than the intervention alone. Future iterations could use a delayed or alternate-form posttest to better isolate the simulation's specific contribution. Additionally, our assessment covered only a single simulated scenario and did not test transfer of these skills to novel toxicologic emergencies. Finally, the evaluation focused exclusively on immediate knowledge acquisition and does not address long-term retention or transfer to clinical practice.

We plan to continue this simulation in our clinical toxicology elective. Our institution also incorporated the valproic acid lecture and quiz into a 2-week online toxicology elective, running a similar pretest-posttest format with the lecture as the sole intervention. Preliminary data from that lecture-only cohort also show a statistically significant improvement in knowledge scores. However, that curriculum administers the pretest immediately before the lecture rather than after the 2-week interval used here, so the two results are not directly comparable, and this finding should not be interpreted as evidence that lecture alone is sufficient to produce the gains seen in this study. We plan to expand this comparison to short- and long-term assessments, and to extend the simulation into emergency medicine and neurology training at Virginia Commonwealth University Health (VCU Health), including residents and varied patient presentations to practice activated charcoal administration, hemodialysis, central and arterial line placement, and airway management.

## Appendices


Stations and Equipment.docxPretest.docxPresimulation Questionnaire.docxValproic Acid Lecture.pptxSimulated Case.docxLaboratory Values and Imaging.docxCritical Actions Checklist.docxValproic Acid Debrief.pptxPosttest with Answers.docxPostsimulation Questionnaire.docx
*All appendices are peer reviewed as integral parts of the Original Publication.*
